# Bariatric Surgery as Potential Treatment for Nonalcoholic Fatty Liver Disease: A Future Treatment by Choice or by Chance?

**DOI:** 10.1155/2013/839275

**Published:** 2013-01-29

**Authors:** Shuja Hafeez, Mohamed H. Ahmed

**Affiliations:** ^1^Department of Emergency Medicine, The James Cook University Hospital, Middlesbrough TS4 3BW, UK; ^2^Department of Medicine, Wexham Park Hospital, Slough, Berkshire SL2 4HL, UK

## Abstract

Morbid obesity is strongly associated with nonalcoholic fatty liver disease (NAFLD) which is one of the most common causes of chronic liver disease worldwide. The current best treatment of NAFLD and NASH is weight reduction through life style modifications, antiobesity medication, and bariatric surgery. Importantly, bariatric surgery is the best alternative option for weight reduction if lifestyle modifications and pharmacological therapy have not yielded long-term success. Bariatric surgery is an effective treatment option for individuals who are grossly obese and associated with marked decrease in obesity-related morbidity and mortality. The most common performed bariatric surgery is Roux-en-Y gastric bypass (RYGB). The current evidence suggests that bariatric surgery in these patients will decrease the grade of steatosis, hepatic inflammation, and fibrosis. NAFLD *per se* is not an indication for bariatric surgery. Further research is urgently needed to determine (i) the benefit of bariatric surgery in NAFLD patients at high risk of developing liver cirrhosis (ii) the role of bariatric surgery in modulation of complications of NAFLD like diabetes and cardiovascular disease. The outcomes of the future research will determine whether bariatric surgery will be one of the recommended choice for treatment of the most progressive type of NAFLD.

## 1. Introduction

Nonalcoholic fatty liver disease (NAFLD) is an increasingly recognised condition that occurs in all age groups and ethnicities [1]. Although first recognised in the 1930s [2], acknowledged clinically in the 1950s [3], and characterised histopathologically in the 1980s [4], only recently has the growing burden of disease gained notoriety [5]. Over this time, an assortment of terms has been used to represent this curiosity, including fatty liver hepatitis, nonalcoholic Laennec's disease, diabetes hepatitis, alcohol-like liver disease, steatonecrosis, and nonalcoholic steatohepatitis [6, 7]. NAFLD has become the term of choice and describes a broad spectrum of hepatic conditions, which vary histologically. At one end of the spectrum lies simple steatosis, a relatively benign condition [8], in which there is an accumulation of lipids within the hepatocytes >5% of liver weight [1]. At the other end is nonalcoholic steatohepatitis (NASH), which is steatosis in the presence of necroinflammatory hepatocellular changes [9]; this subset of patients is at increased risk of further progressing to fibrosis [10], cirrhosis [11], hepatocellular carcinoma [12], and terminal liver failure [13].

The diagnosis of NAFLD is often made fortuitously; steatosis and NASH are clinically indistinguishable [14] and in the absence of hepatic decompensation, largely asymptomatic [6]. Some patients may complain of malaise or right upper quadrant pain, with hepatomegaly being the only clinical observation [7]. Those at the malign end of the NAFLD spectrum may demonstrate evidence of cirrhosis, bruising, varices, ascites, splenomegaly, jaundice, and encephalopathy [15]. Identification of patients before they reach this point is therefore important, to avoid liver-related morbidity and mortality. A high index of clinical suspicion is needed to diagnose NAFLD, particularly in those whose serum liver function tests reveal an elevated ALT [16]. Meticulous history taking and investigation is also needed to exclude excess alcoholism (>10 g ethanol per day [5]) and other causes of liver disease or steatosis, viral, nutritional, drug/environmental/toxin induced, metabolic, genetically inherited, bowel diseases, and endocrine [6, 17–19].

Noninvasive imaging techniques such as ultrasound (US), magnetic resonance imaging (MRI), and computed tomography (CT) can be employed to further investigate the liver, as all are able to detect NAFLD [7]. However, negative imaging studies do not rule out NAFLD [40]. Furthermore, these imaging techniques are unable to differentiate between simple benign steatosis, NASH, and the degree of fibrosis [41]; they merely describe the presence of a “fatty liver.” Thus, liver biopsy remains the gold standard for the confirmation and staging of NAFLD [1], but it is not without its risks [42], and the decision to proceed with biopsy must be individualised and involves the advantage and disadvantages of biopsy. NAFLD is not a condition that occurs in isolation. It is now known to have strong associations with insulin resistance [6], diabetes mellitus [43], dyslipidaemia [44], hypertension [45], and central obesity [46], all of which are components of the metabolic syndrome. In light of these associations, NAFLD is often regarded as the hepatic manifestation of the metabolic syndrome [13, 42].

Indeed the prevalence of NAFLD continues to grow [47] and appears to mirror the rising trends in obesity and type 2 diabetes in those subscribing to an increasingly sedentary lifestyle [10]. In the adult population, NAFLD is thought to affect up to 24% of individuals [6], and in the obese this figure rises to up to 74% [48]. This trend is also mirrored in the children population; up to 2.6% of the general population [49] and up to 52.8% of obese children [50] have NAFLD. Approximately 5% of those with NAFLD will progress to cirrhosis with nearly 2% will die from complications stemming from a cirrhotic liver [51]. Individuals with NAFLD are also at a higher risk of all causes of mortality [1], largely due to the coexistence of metabolic syndrome [52].

The current mainstays of treatment aim to reduce weight [5] and ameliorate the metabolic disturbances associated with the metabolic syndrome [1]. Although no drugs are specifically licensed for the treatment of NAFLD, there is evidence to support the use of selected agents. Antiobesity medications such as Orlistat [53] and drugs that augment insulin sensitivity and reduce plasma glucose concentrations and oxidative stress such as Thiazolidinediones [54] and Metformin [52] are amongst those shown to improve liver histology in NAFLD. Alongside the pharmacological approach to management run conservative measures, such as weight loss through dieting, exercise, and lifestyle modification, all of which have shown to improve liver histology in both adults [55] and children [56] with NAFLD. Rapid weight loss was thought to adversely affect the liver in NAFLD [57] and this was thought to be due to deficiency of macro/micronutrients [1].

Bariatric surgery has been shown to be superior to conservative measures with respect to weight reduction in the obese [58]. It can lower the long-term morbidity of obesity by up to 40% [59] and alleviate the myriad of illnesses associated with it [60]. Since obesity is the key cause of NAFLD, this paper will look at bariatric surgery as a means of treatment for NAFLD. Worldwide, the number of surgical bariatric procedures has risen by an estimated 761% over the past ten years [61]. Procedures can be divided into 3 broad categories based on their mechanism of action [62]:

1. restrictive procedures, which aim to restrict the amount of food that can be eaten by surgically reducing the size of the stomach. The restrictive procedures most commonly performed are vertical banded gastroplasty, laparoscopic adjustable gastric banding, and sleeve gastrectomy;
2. malabsorptive procedures are less popular than restrictive procedures as they are more technically demanding to be performed and patients often develop nutritional deficiencies. Procedures aim to bypass a segment of the small bowel so that less food is absorbed (biliopancreatic diversion, biliopancreatic diversion with duodenal pouch);
3. hybrid procedure aims to restrict food intake by creating a small gastric pouch which also limits absorption by bypassing the proximal small bowel—the Roux-en-Y gastric bypass (RYGB). It leaves 95% of the small bowel intact and so avoids many of the unwanted malabsorptive side effects such as diarrhoea and nutritional deficiencies. This is the most common surgical bariatric procedure performed.

## 2. Potential Benefit of Bariatric Surgery as Treatment of NAFLD

There is a large body of evidence to support the fact that when performed by skilled surgeons, bariatric surgery is safe [63], effective in reducing weight [64], improves quality of life [65], decreases obesity related disease [66], and increases life expectancy [67]. Despite this, at present there is currently a lack of randomised controlled trials examining the effects of bariatric surgery on NAFLD; the only available studies are either retrospective or prospective cohort studies. We have searched the Medline for studies between 1970 and 2012 that looked at the impact of bariatric surgery on NAFLD. We have identified twenty-two studies and their findings were reviewed and presented in the subsequent discussion and categorised according to surgical technique implemented (Tables 1, 2, 3, and 4). In addition, we included a section about the pathophysiological changes that take place following bariatric surgery that potentially contribute towards the treatment of NAFLD.

### 2.1. The Roux-en-Y Gastric Bypass

Considerable studies showed that RYGB is associated with marked improvement in NAFLD. For instance, twelve studies [20–31] (five retrospective and seven prospective) (Table 1) with sample sizes ranged from 7 to 116 participants and the follow-up period after surgery varied from 12 to 32 months, with one study having an unidentifiable follow-up period [30]. The predominant findings across these studies beside successful weight loss were a histological improvement in steatosis, inflammation, and fibrosis following Roux-en-Y gastric bypass. However, five of the studies [20, 22, 23, 26, 28] had reported a few cases of worsening or new fibrosis in some of the study participants after RYGB. This could be attributed to these patients already having a pre-existing degree of fibrosis which subsequently worsened by the surgical intervention, or due to a lack of adequate replacement of macro/micronutrients. The earliest of these studies, conducted by Silverman et al. [20], found post-RYGB in 91 of the obese individuals selected for the study; 83 showed improvement in steatosis, 5 showed no change, and 3 had increased steatosis. Pre-RYGB biopsies revealed that 13 participants had perisinusoidal fibrosis; this was eliminated or reduced in all but 2 individuals, who showed no change in fibrosis following RYGB. One study participant developed new perisinusoidal fibrosis within the follow-up period.

Barker et al. [25] found that weight loss achieved through RYGB improved liver histopathology in 17 out of the 19 obese patients selected for the study. In all 17 patients the initial histopathological criteria for NASH were no longer met on follow-up biopsy. As well as NASH resolution, subjects also displayed weight loss and an improvement in biochemical markers of metabolic syndrome, triglycerides, lipoprotein, and fasting glucose. Fibrosis was found to have worsened in 2 of the 19 patients. In the study by Csendes et al. [26], RYGB resulted in weight loss in all 16 of the study participants, all but 1 had abnormal liver histology prior to the procedure. The solitary participant with normal preoperative liver histology remained normal on follow-up biopsy, 11 had returned to normal liver histology, 2 showed histological improvement, 1 participant had progressed to fibrosis, and 1 participant who had cirrhosis prior to RYGB continued to have cirrhosis.

Moretto et al. [31] evaluated liver biopsies taken during RYGB surgery and after weight loss in 78 morbidly obese individuals. 35 of the 78 participants had fibrosis on first biopsy; after weight loss only 19 of these were found to have fibrosis. Of the 43 individuals to be fibrosis free on initial biopsy, 5 were found to have developed fibrosis on follow-up biopsy after the RYGB procedure. Liu et al. [29] demonstrated that RYGB surgery resolved NASH in all 23 of the 39 patients whom displayed evidence of NASH on initial biopsy. One patient developed fibrosis following RYGB, but no participant showed worsening of existing fibrosis or cirrhosis.

Studies by Clark et al. [21] and de Almeida et al. [27] both found that RYGB surgery led to an improvement in liver steatosis, inflammation, and fibrosis in the vast majority of study participants on follow-up biopsy. Neither study showed evidence of worsening histopathology after RYGB. Similarly, Weiner [30] demonstrated that using one of three bariatric surgical measures (RYGB or adjustable gastric banding and biliopancreatic diversions with duodenal switch) resulted in an improvement in all obesity-related comorbidities, as well as a complete regression of NAFLD in 83% of the study cohort. He concluded that obesity surgery successfully improves hepatic steatosis, inflammation, and fibrosis with no evidence to suggest worsening.

Furuya et al. [28] discovered that two years after RYGB in 18 morbidly obese patients, all of whom had some degree of NAFLD, a mean excess weight loss of 60% was observed as well as an elimination of steatosis in 84% and an elimination of fibrosis in 75% of patients in the study. Furthermore, obesity-related comorbidities also improved and there was no evidence to suggest that RYGB led to worsening hepatic histology. The study looking at the effects of RYGB on NAFLD with the smallest cohort of study participants was conducted by Klein et al. [24]. They reported that RYGB surgery normalises the metabolic abnormalities involved in the pathogenesis of NAFLD and decreases the expression of hepatic factors involved in the progression of liver fibrosis and inflammation. Mottin et al. [23] focused on the changes in histological hepatic steatosis in 90 morbidly obese patients when comparing biopsies taken during RYGB and 1 year after surgery. They found that 82% showed improvement in steatosis with no patient showing a worsening in histology—hepatic fibrosis was not measured in this study.

Mattar et al. [22] studied 70 patients who underwent one of three bariatric surgical weight loss operations; 59% had RYGB, 9% had adjustable gastric banding, and 33% had sleeve gastrectomy—a restrictive procedure that permanently reduces the size of the stomach to ~15 mL [68]. A liver biopsy was taken at the point of surgery and a follow-up biopsy was taken 15 ± 9 months later. They showed a reduction in the prevalence of metabolic syndrome from 70% to 14% and an improvement in liver steatosis, inflammation, and fibrosis, with inflammation and fibrosis resolving completely in 37% and 20% of patients, respectively. Interestingly, another conclusion drawn from this study was that the RYGB group lost greater excess weight and a greater improvement in the grade of liver disease when compared to the two restrictive procedures. No participant in this study showed evidence of worsening liver histology on follow up biopsy (Table 1). 

### 2.2. Vertical Band Gastroplasty

Of the 4 studies found looking at the effects of vertical band gastroplasty (VBG) on NAFLD, the smallest was conducted by Ranlov and Hardt [32], consisting of 15 patients in total of which 8 underwent VBG. After one year there was a significant regression of hepatic steatosis and the occurrence of steatosis had fallen from 73% to 40%—none of the cohort exhibited any evidence of fibrosis at any point in the study. Jaskiewicz et al. [33] also demonstrated an improvement in inflammation and steatosis following VGB in the absence of any new fibrotic changes. 

The earliest of these studies was carried out by Luyckx et al. in 1998 [48]. Steatosis was found to be markedly reduced following weight loss; 45% of the 69 participants had normal hepatic biopsies on followup (versus 13% on initial biopsy) and the severity of steatosis was also reduced in those in whom steatosis had persisted. Despite these encouraging findings, an increase in the incidence of hepatocellular inflammation was noted on repeat biopsy, from 14% to 26%. In a similar but more recent study, Stratopoulos et al. [34] found in their cohort of 51 morbidly obese individuals undergoing VGB that there was a significant improvement in steatosis and steatohepatitis after weight loss following bariatric surgery. Although an overall decrease in fibrosis was observed on follow-up biopsy, 11.7% had increased fibrosis. Indeed it appears that VGB has fallen out of favour in recent years; a recent prospective study comparing forms of restrictive bariatric procedures [69] found that VGB had a failure rate of 65%, with 60% of patients eventually requiring conversion to RYGB (Table 2). 

### 2.3. Adjustable Gastric Banding

Adjustable gastric banding (AGB) is the second most frequently performed bariatric surgery worldwide [70]. It has been proven to be more effective than lifestyle change, pharmacotherapy, low calorie diets, and behavioural modification in long-term sustainable weight loss in moderately obese patients [71]. Only three studies were identified that examined the effects of this mode of bariatric surgery on NAFLD (Table 1). 

Dixon et al. [35] conducted paired liver biopsies on 36 selected obese patients, the first at the time of AGB placement and the second after weight loss. Initial biopsies revealed that inflammation was present in 23 patients and steatosis in 12. On follow-up biopsy, taken after 25.6 ± 10 months, there were improvements in steatosis, inflammation, and fibrosis, with only 4 patients fulfilling the criteria for NASH. There were no reports of any worsening histology. The same author led a second similar investigation in 2006 [36], in which baseline histological examination revealed that 30 out of 60 morbidly obese individuals had evidence of NASH. On follow-up biopsy, taken 29.5 ± 10 months after AGB, only 10% displayed NASH, with an improvement in steatosis, inflammation, and fibrosis seen in all as well as improvements in biochemical markers of liver function. 

AGB was one of three interventions used by Mathurin et al. [37], the other two being RYGB and biliointestinal bypass, to study the long-term effects of bariatric surgery on NAFLD. Although the percentage of patients with steatosis fell from 37.4% to 16% after surgery, inflammation remained unchanged and a significant increase in fibrosis was seen in 20% of patients. There was no significant difference between the three surgical groups, and those who progressed to fibrosis also became more insulin resistant. They also found a positive correlation between the presence of hepatic steatosis/ballooning and insulin resistance (Table 3). 

### 2.4. Malabsorptive Procedures

Only two studies were found that utilised malabsorptive bariatric surgical techniques to examine the effects on NAFLD [38, 39]. Kral et al. [38] performed liver biopsies on biliopancreatic diversion and then on followup 41 ± 25 months later on 104 patients. Biopsies were graded on the basis of steatosis, fibrosis, and inflammation by a blinded hepatopathologist. Steatosis grades decreased in correlation with weight loss as expected, and as seen in previous studies. However, they observed a postoperative increase in fibrosis in 40%, a decrease in 27%, and no change in 33%. The changes in fibrosis were related to initial fibrosis grades; those with a higher grade of fibrosis on first biopsy saw a reduction, and those with a lower baseline grade saw an increase. Eighteen of the initial cohort showed resolution of mild inflammation, whereas 10 patients developed new mild inflammation. 

Keshishian et al. [39] studied repeat liver biopsies on 78 patients at the time of, and 6–36 months after duodenal switch (DS) operation. Liver function tests had worsened by the 6-month period, but normalised by 12 month, and remained normal thereafter. Furthermore, hepatic inflammation had also slightly worsened at the 6-month mark, but then improvements were seen at and beyond 12 months. This initial worsening was attributed to the rapid weight loss experienced early in patients undergoing bariatric surgery. By three years, the histological degree of steatosis had improved by 60% and the severity of inflammation improved by 3 grades. They concluded that no hepatic detrimental effects are seen beyond 6 months; however, fibrosis was not examined in this study (Table 4).

## 3. Bariatric Surgery and Metabolic Syndrome

Several studies have shown benefit of utilising bariatric surgery as a mode of treatment for metabolic syndrome and its components. To et al. [72] examined the effects of sleeve-gastrectomy, a restrictive procedure which permanently reduces the size of the stomach, on 52 morbidly obese patients who fulfilled the criteria for metabolic syndrome. Postoperative followups were conducted at 6, 12, and 24 months. They found improvements in all features of metabolic syndrome; a reduction in triglycerides, hypertension, obesity and fasting glucose as well as an increase in HDL cholesterol. The metabolic improvements did not correlate with the degree of weight loss, and the most drastic improvements were observed within the first 6 months following surgery, when significant weight reduction occurred.

A recent Brazilian study [73] specifically looked at the relationship between BMI and metabolic syndrome following Roux-en-Y gastric bypass. In the cohort of 149 patients there was a significant resolution of metabolic syndrome postoperatively on the 180-day follow-up assessment. A later Brazilian study conducted by Júnior et al. [74] looked at 35 patients who underwent the RYGB; 27 of these had a diagnosis of metabolic syndrome. When these patients were followed up 34.4 ± 15 months after surgery, not only was a reduction in BMI observed, only 2 patients fulfilled the criteria for metabolic syndrome. Improvements in abdominal circumference, fasting glucose, blood pressure, HDL-cholesterol, and triglycerides were also seen. Inabnet III et al. [75] searched “The National Database for the American Society for Metabolic and Bariatric Surgery Centre of Excellence Program” for patients with metabolic syndrome that were undergoing bariatric surgery between 2007 and 2010. Of this group of 23,106 patients, 62% underwent RYGB, 32% had gastric banding, 4.5% had sleeve gastrectomy, and only 1.5% had biliopancreatic diversion with duodenal switch. Despite the fact that the metabolic syndrome group had a higher prevalence of postoperative complications, there was an improvement in their comorbid state.

Bretón et al. [76] looked at 46 morbidly obese patients undergoing laparoscopic bypass surgery, of which twenty-eight fulfilled the criteria for metabolic syndrome preoperatively. On follow up 2 years after surgery, they found resolution of hypertension, dysglycaemia, and dyslipidaemia in 85%, 93.8%, and 95.6% of patients, respectively. Interestingly, Pontiroli et al. [77] compared the long-term effects of restrictive (gastric banding) and malabsorptive (biliopancreatic diversion) bariatric procedures on metabolic syndrome with a control group whom utilised conservative measures (diet and lifestyle adaptations). They found that the studied parameters of diabetes, hypertension, and metabolic syndrome disappeared more in the surgical group than the control. Biliopancreatic diversion was found to be more effective than gastric banding in reducing BMI, and neither surgical group showed any new diagnoses of metabolic syndrome, whereas the control group did, suggesting that surgery is a more successful mode of treatment for metabolic syndrome.

The Surgical Department of the Mayo Clinic [78] carried out a retrospective study between 1990 and 2003 on 337 patients evaluated for bariatric surgery. Two groups were established; 180 undergoing RYGB (156 had metabolic syndrome) and 157 nonoperative patients (133 had metabolic syndrome) enrolled in a weight reduction program. After a mean followup of 3.4 years, all components of metabolic syndrome improved in the surgical group; there was a reduction in the prevalence of metabolic syndrome from 87% to 29%, while nonoperative patient group also showed a decrease in the prevalence of metabolic syndrome, from 85% to 75%. These findings suggest that RYGB induces a considerable and persistent improvement in the prevalence of metabolic syndrome. 

Nugent et al. (2008) [79] assessed the impact of bariatric surgery on metabolic syndrome, by looking at 286 patients; 160 underwent restrictive procedures, 78 had malabsorptive, and 48 had a combination procedure. After 297 ± 271 days, 263 patients were followed up. Prior to surgery 40% had metabolic syndrome, compared to only 10% on followup. Furthermore, all components of metabolic syndrome also improved; waist circumference, BMI, fasting serum triglycerides, and fasting glucose. The prevalence of type 2 diabetes also fell from 30% to 10%. Madan et al. [80] found in their study that the prevalence of metabolic syndrome fell from 60% to 2% in their cohort of 53 patients undergoing laparoscopic gastric bypass surgery. Lee et al. [81] found metabolic syndrome to be present in 52% of morbidly obese individuals enrolling in a bariatric surgery program, and on 1-year followup, there was a resolution rate of 95.6% of the condition. A growing body of evidence also exists to demonstrate that an improvement in the components of metabolic syndrome also takes place following bariatric surgery [82–85]. Carson et al. [86] reported that there was an improvement or complete resolution of hypertension in 70% of patients undergoing gastric bypass surgery. Two studies looking specifically at dyslipidaemia found a resolution in hyperlipidaemia in morbidly obese patients following bariatric surgery, which was maintained at 1-year followup [87] and 5-year follow-up [88]. 

These findings of resolution of the components of metabolic syndrome, and improvement in the condition itself following bariatric surgery, are clearly indicative that currently bariatric surgery is an effective and safe surgical treatment option for this syndrome.

## 4. Pathophysiological Changes with Bariatric Surgery That Have the Potential to Treat NAFLD

**Figure 1:**
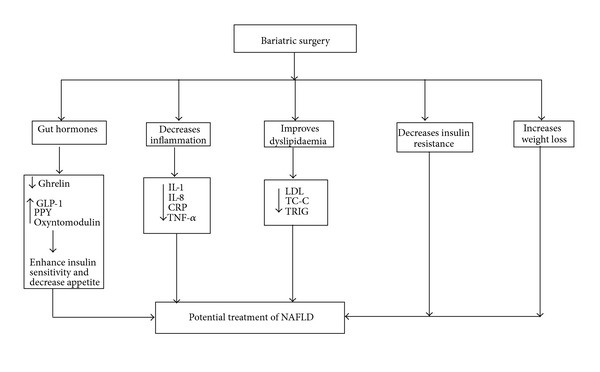
Schematic figure illustrating the complex potential factors that associated with bariatric surgery that may have the potential role in the treatment of NAFLD.

The full understanding of the pathogenesis behind NAFLD is not yet established and probably involves complex factors that alter different metabolic events (Figure 1). The key pathognomonic feature of pathogenesis of NAFLD is insulin resistance and dyslipidaemia. Obesity and central obesity are associated with an increase in free fatty acid supply to the liver and ultimately insulin resistance [89–91]. Excessive consumption of glucose or sucrose is also shown to promote NAFLD due to the increase of de novo lipogenesis. In addition, fatty food may also precipitate NASH. There is a strong link between insulin resistance and excessive deposition of triglyceride in the hepatocytes, which is the hallmark for diagnosis of NAFLD. The excessive/ectopic fat deposition in the liver could be due to increased fatty acid delivery from adipose tissue, increased synthesis of fatty acid via the de novo pathway, increased dietary fat, decreased mitochondrial oxidation, decreased clearance of VLDL particles, or these factors in combination [5, 6].

Fat tissue is now considered a metabolically active endocrine organ producing proinflammatory cytokines including TNF-*α*, IL-6, and IL-8, and there is evidence to support the activation of other inflammatory pathways, oxidative stress, and the de novo pathway by TNF-*α*. Inflammation is associated insulin resistance resulting in increased lipolysis in adipose tissue, increased NEFA uptake by hepatocytes, and increased triglyceride synthesis in the liver. As a consequence of abnormal fat accumulation in the hepatocytes, there is marked derangement in the insulin signalling pathways in the liver [11]. Adiponectin has been shown to decrease de novo fatty acid synthesis and enhance fat oxidation, with levels of adiponectin increasing after dietary fat ingestion [92]. Decreased adiponectin is associated with insulin resistance and hyperlipidaemia and low level of adiponectin was shown in NAFLD independent of the components of the metabolic syndrome [93].

Bariatric surgery is likely to have potential benefit in ameliorating the following factors that contribute in marked way to the pathogenesis of NAFLD.

- * Insulin resistance*. One of the immediate benefits of bariatric surgery before weight loss is remission of type 2 diabetes and improvement of insulin sensitivity. Pories et al. showed that in 608 obese with type 2 diabetes, 83% have normoglycaemia before weight loss but within days of RYGB [94]. A meta-analysis of 136 bariatric surgery studies including 22094 individuals confirmed an overall 84% remission of type 2 daibetes after RYGB [95]. Interestingly, two large multicentre studies showed similar outcomes in treating type 2 diabetes with one study showed 92% decrease in diabetes [60, 61].
- *Dyslipidaemia.* Several studies showed that bariatric surgery is associated with significant improvement in the lipid profile. For instance, accumulating body of evidence showed that bariatric surgery is associated with marked decrease in LDL-c, triglyceride, and lipoprotein (A). Furthermore, bariatric surgery is not only associated with stopping lipid lowering medication but also stopping antihypertensive medication [86–88].
- *Inflammation*. Obesity is associated with low-grade chronic inflammation and adipose tissue is a main source of excess production of cytokines like tumour necrosis factor-*α* (TNF-*α*), interleukin-18 (IL-18), IL-1, IL-8, monocyte chemoattractant protein-1, and C reactive protein (CRP) [96]. Excess production of TNF-*α* and low adiponectin are associated with insulin resistance and nonalcoholic fatty liver [5]. Interestingly, IL-18 has the capacity to stimulate the secretion of TNF-*α*. Furthermore, TNF-*α* plays crucial role in the pathogenesis of NAFLD. Bariatric surgery has been shown to ameliorate insulin resistance, improve the adiponectin level, and decrease IL-18, CRP, and TNF-*α*. Interestingly, RYGB in five individuals with diabetes was associated with a decrease of CRP and leptin with no alteration in the level of adiponectin and TNF-*α* [96, 97]. Therefore, it is possible to suggest that bariatric surgery has the potential benefit of treating obesity and decreasing the low-grade associated inflammatory state.
- *Adiponectin.* Is known to be antiatherogenic, anti-inflammatory, and antidiabetogenic and is decreased with increasing fat mass, BMI and serum triglyceride. A low adiponectin level is linked to insulin resistance, type 2 diabetes, atherosclerosis, and acute coronary syndrome [92, 93].
- *Weight loss*. Currently the only effective treatment for NAFLD is weight loss. There is an agreement in the literature that the majority of well performed bariatric surgery is associated with sustained and significant weight loss [21–31].
- *Intestinal hormones.* Bariatric surgery (RYGB) is associated with a decrease in Ghrelin, which is known to; stimulate insulin counter regulatory hormones, decrease adiponectin, and block hepatic insulin signalling. Furthermore, RGYB is associated with an increase in glucagon like peptide-1 (GLP-1) which subsequently enhances glucose tolerance by enhancing insulin secretion, suppressing glucagon production, inhibiting gastric emptying, and increasing B-cell mass. Other hormones that suppress appetite and produced in excess with bariatric surgery are peptide YY (PYY) and Oxyntomodulin [98].

## 5. Conclusion

The worldwide rising obesity pandemic is paralleled by rising rates of the metabolic syndrome and its hepatic manifestation, NAFLD. The therapeutic options for the treatment of obesity and NAFLD include lifestyle adjustments, pharmacotherapy, and surgical interventions. With regards to sustained weight loss, in those who adopt lifestyle changes such as dieting, 33–66% subsequently regain more weight than lost whilst dieting. Similarly, pharmacotherapy results in a significant prolonged weight loss; however, this only lasts as long as the medication is taken, as weight gain is often observed once the medication has ceased.

Bariatric surgery appears to show more promise than its noninvasive counterparts. Subjects experience a 40–71% loss of excess body weight following surgery, and weight loss is sustained. Importantly, weight loss is one of the first line recommendations to individuals with NAFLD. RYGB is associated with complete remission of type 2 diabetes, suggesting greater potential benefit in treating NAFLD. The mechanism of how bariatric surgery plays a role as potential treatment of NAFLD is complex and not fully understood (Figure 1). It is possible to suggest that in addition to weight loss, bariatric surgery normalizes insulin resistance and decreases dyslipidaemia and inflammation. The alteration in gut hormone production following bariatric surgery and its role in insulin sensitivity has generated a lot of interest. Urgent large scale clinical studies are needed to further evaluate the role of bariatric surgery as a viable option for the treatment for NAFLD, in the obese population.

## Figures and Tables

**Table 1:** Considerable studies showed that RYGB is associated with marked improvement in NAFLD.

Roux-en-Y					
Study	Ref	Main outcomes	Type of study	Sample size	Followup
Silverman et al., 1995	[20]	Improved steatosis and fibrosis	Retrospective cohort	91	18.4 months
Clark et al., 2005	[21]	Improved steatosis, fibrosis, and inflammation	Prospective cohort	16	305 ± 131 days
Mattar et al., 2005	[22]	Improved metabolic syndrome, steatosis, and fibrosis	Prospective cohort	70	15 ± 9 months
Mottin et al., 2005	[23]	82% improvement in liver steatosis and fibrosis not measured	Retrospective cohort	90	12 months
Klein et al., 2006	[24]	Decreased factors lead to liver fibrosis and inflammation	Prospective cohort	7	12 months
Barker et al., 2006	[25]	Improved histology of NAFLD	Prospective cohort	19	21.4 months
Csendes et al., 2006	[26]	Improved histology in 80%	Prospective cohort	16	22 months
de Almeida et al., 2006	[27]	Improved steatosis, fibrosis, and inflammation	Prospective cohort	16	23.5 ± 8.4 months
Furuya et al., 2007	[28]	Improved steatosis and fibrosis	Prospective cohort	18	24 months
Liu et al., 2007	[29]	Resolved NASH in 60%	Retrospective cohort	39	18 months
Weiner 2010	[30]	Complete regression of NAFLD in 83%	Retrospective cohort	116	18.6 ± 8.3 months
Moretto et al., 2012	[31]	Resolved fibrosis in 50%	Retrospective cohort	78	Unavailable

**Table 2:** Summary of studies of VBG and their effect on NAFLD.

Vertical band gastroplasty					
Study	Ref	Main outcomes	Type of study	Sample size	Followup
Ranløv and Hardt 1990	[32]	Decrease steatosis from 73% to 40%	Prospective cohort	8	12 months
Jaskiewicz et al., 2006	[33]	Improved steatosis and inflammation	Prospective cohort	10	8 months
Stratopoulos et al., 2005	[34]	Improved steatosis and NASH	Prospective cohort	216	18 ± 9.6 months

**Table 3:** Summary of studies of AGB and their effect on NAFLD.

Adjustable gastric banding studies					
Study	Ref	Main outcomes	Type of study	Sample size	Followup
Dixon et al., 2004	[35]	Improved steatosis, inflammation, and fibrosis	Prospective cohort	36	25.6 months ± 10 months
Dixon et al., 2006	[36]	Improved steatosis, inflammation, and fibrosis	Prospective cohort	60	29.5 months ± 16 months
Mathurin et al., 2009	[37]	Improved steatosis and significant increase in fibrosis	Prospective cohort	381	50 months ± 7.8 months

**Table 4:** Summary of studies of malabsorptive procedure and their effect on NAFLD.

Malabsorptive procedure					
Study	Ref	Main outcomes	Type of study	Sample size	Followup
Kral et al., 2004	[38]	Postoperative increase in fibrosis in 40%, a decrease in 27%, and no change in 33%	Prospective cohort	104	41 ± 25 months
Keshishian et al., 2005	[39]	Significant improvement in steatosis and inflammation	Retrospective cohort	78	6–36 months
